# ‘Recombining’ biological motherhoods. Towards two ‘complete’ biological mothers

**DOI:** 10.1136/jme-2023-109610

**Published:** 2024-05-02

**Authors:** Emanuele Mangione

**Affiliations:** 1 Medicine and Surgery, University of Insubria, Varese, Italy

**Keywords:** Feminism, Reproductive Medicine, Women, In Vitro Techniques, Fertilization in Vitro

## Abstract

Within feminist literature from the early 1970s to this day, assisted reproductive technologies have been largely known to divide, replace or eliminate biological motherhood. For example, while in the past biological motherhood was considered a continuous experience, in vitro fertilisation (IVF) and IVF using egg donation allowed a split between two biological mothers, one providing eggs (genetic mother) and the other one gestation (gestational mother). This split was considered irreparable: the genetic mother could not be also gestational, and vice versa. On the contrary, this paper aims to show that assisted reproductive technologies may also have a constructive potential towards biological motherhood(s). To explain how it could be possible, two existing techniques are explored: the first is maternal spindle transfer, which allows a double genetic motherhood; the second is reciprocal effortless IVF, which supposedly enables a double gestational motherhood. While in the first part, these techniques are examined singularly, in the second part a feasible combination of them is speculated. The idea is that assisted reproductive technologies could ‘recombine’ genetic and gestational motherhood in two figures that include both, namely in two ‘complete’ biological mothers, both genetic and gestational.

## Introduction

 Before the introduction of in vitro fertilisation (IVF) and egg donation in the second half of the 20th century, biological motherhood was described as a continuum that began when a woman conceived an entity and, passing through gestation, ended when she delivered it.^1^ While biological fatherhood was described as a discontinuous process because men were typically alienated from the seed that they delivered,^2 3^ women experienced no alienation from their eggs because of their capacity to gestate.^4^ Women’s reproductive process was therefore represented as a unitary, unified or total biological process that provided them ‘reproductive continuity’.^1^ As a result, the mother was said to be always certain because she could be easily identified with the one who gave birth, and there was no doubt that the one who gave birth to an entity was the same who had conceived and gestated it.^5^ A child could only have a single biological mother, and that was perceived so true and ‘natural’ that, at least until the development of assisted reproductive technologies, biological motherhood was not much questioned.^6^

Since the birth of the first baby with IVF in 1978 and the first successful pregnancy with donated eggs in 1983, assisted reproductive technologies changed reproduction and its public perception in that they caused a split not only between biological and social parenthood, as in adoption or traditional surrogacy, but also within biological parenthood itself; most notably, within biological motherhood.^7^ Let one consider IVF using egg donation: egg donation is a practice in which an individual (the donor) provides her egg to allow another individual (the recipient) to become pregnant. Before its introduction, there could be only a biological ‘mother’ because the one who conceived an entity was the same who gestated and delivered it. With egg donation, a child could have not one, but *two* biological mothers instead: a genetic mother, who is ‘the donor of the egg’,^8^ and a gestational mother, ‘who bears and gives birth to the baby’.^9^ Because of egg donation and the following split between a genetic motherhood and a gestational motherhood, the reproductive continuity that biological motherhood provided women had been broken, and that could not but influence biological motherhood.

Whether they are seen as good, evil or a ‘double-edged sword’,^10^ assisted reproductive technologies are still widely recognised to have altered biological motherhood to the point that it is claimed to have been split,^11^ divided,^12^ fractioned,^13^ compartmentalised,^6^ segmented,^2^ deconstructed,^14^ decomposed,^7 15^ impoverished,^2^ degraded,^14^ broken,^16^ fractured^7^ or even ‘torn apart’.^17^ Although the terms used are not fully equivalent, they all emphasise that assisted reproductive technologies have altered both biological motherhood and the way it is societally perceived through the creation of multiple biological motherhoods. Philosophically speaking, with the development of assisted reproductive technologies the biological mother stopped being always certain because a child could have more than one biological mother.^17^ Considering the different nomenclatures that Snowden and Mitchell advanced for mothers,^18^ a mother could be only genetic, only gestational/carrying, or both, not to mention that she could even be a social/nurturing mother. The creation of multiple biological motherhoods following the advancement of assisted reproductive technologies still raises debates on who the ‘true’, ‘real’, ‘prevailing’, or even ‘perfect’ biological mother should be, whether genetic or gestational, and it is often said to have irreversibly affected motherhood.^17 19^ It is common opinion that motherhood as it has been known disappeared or shall disappear since, due to assisted reproductive technologies, it lost or is about to lose its ‘true nature’ or ‘original physiognomy’.^20^ Predictions about the end or the disappearance of motherhood may be explained by two main observations: the first one is that biological motherhood could be split by assisted reproductive technologies into different parts, stopping being unique; the second one, which derives from the first, is that any division in biological motherhood was implicitly assumed to be ‘irreparable’ because—when there was a division between a genetic mother and a gestational mother—the genetic mother could not be also gestational, and vice versa.^15 17^

## Objectives

Within feminist literature from the early 1970s to this day, assisted reproductive technologies have been largely known to divide, replace or eliminate biological motherhood.^7^ Conversely, this paper aims to show that assisted reproductive technologies may also have a constructive potential towards biological motherhood(s), ‘recombining’ it in new forms. ‘Recombining’ biological motherhood here means bringing its two separated dimensions—genetic motherhood and gestational motherhood—together again in two ‘complete’ biological mothers. According to Snowden and Mitchell, a mother is ‘complete’ when she is genetic, gestational and social at the same time^18^; with only reference to biological motherhood, it follows that a biological mother can be considered ‘complete’ when she is both genetic and gestational. Recombining biological motherhoods means, in other words, joining genetic and gestational motherhood in two figures that are both genetic and gestational. This does not mean that a ‘complete’ motherhood should be preferred to other kinds of motherhood, or that all people will seek it. Not only could the use of some assisted reproductive techniques be illegal in some countries but also there exist many ways for an individual to perceive themselves as mothers, and I suggest that on no account can a way be deemed ‘preferable’ to or ‘better’ than all the others. Therefore, the term ‘complete mother’ is here used with a descriptive intent and not a normative one.

To show how biological motherhoods could be technologically recombined, two existing techniques are mainly explored: first, a mitochondrial transfer technique known as ‘maternal spindle transfer’ (MST), which causes the conceived to inherit the nuclear DNA of an individual and the mitochondrial DNA of another individual; second, the so-called ‘reciprocal effortless IVF’ (ReIVF), which enables two individuals to have the same entity within both their bodies. While MST enables two individuals to be the ‘genetic mothers’ of the same entity since both contribute genetically to the entity’s conception, there is controversy that ReIVF or any other similar technique allows a double gestational motherhood; hence, it is only supposed to do so. That said, while in the first part MST and ReIVF are examined singularly, in the second part a feasible combination of them is speculated, and the possibility to have two ‘complete’ biological mothers is discussed.

## Using MST: on a double genetic motherhood today

Mitochondria are organelles located in the cell’s cytoplasm that provide cells with energy and have their own genetic material, that is mitochondrial DNA (mtDNA). Since many diseases are caused by mtDNA mutations, some techniques known as mitochondrial transfer techniques have been invented to prevent them. Mitochondrial transfer techniques consist in the replacement of mitochondria in one or more cells with those extracted from other cell(s) and cause the conceived to inherit the nuclear DNA of their mother and their father and the mtDNA of a donor. Since there are different mitochondrial transfer techniques, and it goes beyond the purposes of this paper to explain all of them, this article addresses as an example one of these techniques, that is, MST. MST has been chosen for reasons of convenience since the other techniques present more than one version and could be difficult to explain in few words (eg, Polar Body 1 Transfer), or they could not be combined with techniques like ReIVF in that they necessarily imply, differently from ReIVF, that embryo fertilisation takes place outside the human body (eg, Pronuclear Transfer, Polar Body 2 Transfer). In MST, both the intended mother and the donor first provide their eggs, which are then enucleated. Second, after the nuclear material from both these eggs has been extracted, the enucleated egg of the intended mother and the donor’s nuclear material are discarded. Third, the intended mother’s nuclear material is inserted into the donor’s enucleated egg. As a result, the donor’s enucleated egg would have the nuclear material of the intended mother and the mtDNA of the donor. Finally, that reconstructed egg is fertilised in vitro, and the embryo obtained with that egg is grown in culture, tested in its quality and viability and frozen until it is transferred to the intended mother or a surrogate mother.^21^

To date, mitochondrial transfer techniques have been used with the therapeutic purpose of preventing the birth of children with mitochondrial diseases, but it has been proposed to make them available for non-therapeutic purposes, including for satisfying lesbian couples’ wish to have a double genetic bond with the same entity.^22^ Beyond the legitimacy of such a technical application, the one who provides mtDNA would be considered more than a donor if that came true. In that case, both the members of a couple would not only be the intended mothers of the same entity but also be more easily acknowledged as its genetic mothers since one would provide nuclear DNA while the other mtDNA.

In some countries such as the UK, the law does not recognise the mtDNA contributor as a "mother" or a legal parent since there is only the legal concept of "gestational motherhood".^23 24^ According to Mills, the exclusion of mtDNA contributors from legal parentage is grounded on a hegemonic cultural view centred on the heteronormative ideal of the nuclear family.^25^ Considering genetic motherhood as a concept that is constructed and shaped by subjective views over time,^22^ I suppose here—in contrast with the UK law—that every genetic contributor is a genetic parent and that, with the use of mitochondrial transfer techniques, the mtDNA contributor is a genetic mother. This assumption comes from an understanding of the family as a social construct that can take different forms and of genetic parenthood as something that concerns both nuclear and mitochondrial DNA. There are reasons to support such an understanding: many scholars such as Mills, Appleby, Cavaliere and Palacios-González suggest that the use of mitochondrial transfer techniques not only is ethically acceptable but it could also have a significant impact on both the identity of the future child and the prospective parents’ perceptions, which means that mtDNA contribution is far from being irrelevant.^2125^,^27^

## Using ReIVF: on a double gestational motherhood today

Little is still known about ReIVF as well as other similar techniques. First, the technique requires the use of a special device in which eggs are mixed with the sperm of a donor. Such a device is inserted in the vagina or the womb of an individual (A) for few days or hours, so that fertilisation, incubation and the early embryo development occur within the body of A. Second, the device is extracted from the body of A and, after it has been opened, the obtained embryos are frozen. Third, the embryos are implanted in the womb of another individual (B), who will carry them until childbirth.^28^ What is certain is that, through ReIVF, two individuals are enabled to *have* the same entity in both their bodies, although not simultaneously. According to one of the fertility specialists who performed ReIVF, the prospective parents ‘both physically carried their child together’,^29^ and this assumption is what could be found in all or in almost every journalistic or academic reflection on ReIVF.^30^ Following this assumption, it could be inferred that, since two individuals had or ‘carried’ the same entity within their bodies, both can be considered as ‘gestational mothers’.

Not even one of the major critics of ReIVF, Musio, apparently questions whether it really allows a double gestational motherhood^15^; nevertheless, there are at least two critical points that need to be clarified. First, considering the womb as the privileged place where pregnancy is located, one could argue that only those who had the embryos in both their wombs can be called ‘gestational mothers’. Second, ReIVF does not allow—at least for the moment—an embryo to be implanted in two different bodies, which means that only a member of a couple can have an embryo implanted within her body. The other one has the embryo(s) within her body, but there are serious doubts whether she ‘carries’ them. If we think that pregnancy, that is gestation, starts at embryo implantation, one might conclude that there is no reason to think that an individual who has a not implanted embryo within her vagina/womb is ‘pregnant’ in any meaningful sense. In this perspective, ReIVF does not allow a double gestational motherhood.

While these considerations are understandable, it must be said that pregnancy and its beginning are still a matter of scientific controversy since there is no consensus on a unique definition of pregnancy, nor is there a general agreement on its ‘nature’, its characteristics, its beginning and even the value and the significance that it should be given.^28^ For instance, the relationship between the one who gestates and the one who is gestated has been described in many ways and under many perspectives: while it has philosophically been described as a symbiotic relationship, a parasitic relationship or a dialogue,^31^ from a metaphysical point of view it has been argued that the gestator contains the gestated (Containment View),^32^ or that the gestated is ‘part’ of the gestator (Parthood View),^33^ and so on. This suggests that the concept of ‘pregnancy’ and, consequently, the concept of ‘gestational motherhood’ heavily rely on different cultural models that influence the way they are perceived.

Although the womb may have some relevance to the definition of pregnancy, Watt and McCarthy hold that a pregnancy can occur even when the embryo does not grow in the womb but, rather, in another place like the abdomen since there is “a genuine, albeit to a large degree dysfunctional, form of maternal biological nurture”.^34^ They also add that embryo implantation could be unnecessary to the definition of a pregnancy; in fact, “even preimplantation a woman is pregnant if she has a child in her reproductive tract, even if she is not the genetic mother”.^34^ Their assumption is that pregnancy begins at fertilisation, that is when the sperm enters an egg and forms a zygote, so that “a woman—like any other woman—has procreated (ie, become a mother) once she and the father have conceived. Similarly, like any other woman, she has gestated once she has had an embryo in her reproductive tract and/*or* has had an embryo implant in some part of her body”.^34^ With the use of the disjunctive conjunction ‘or’, they underline that one could be considered ‘pregnant’ when she has an embryo in her reproductive tract, even if it does not attach to her body. Although she is not physically connected to the developing body of the embryo through the placenta, her prenatal mental representations could be somehow full of meaning and therefore need to be further explored, as well as the ways in which she experiences motherhood.

Concerning the definition of ‘gestational mother’ that has been initially offered—an individual “who bears and *gives birth to the baby*”—,^8 9^ it seems that only the one who gives birth could be referred to as a ‘gestational mother’. However, if someone who had an embryo in her vagina or womb for few hours or days cannot be called ‘gestational mother’, what kind of mother would she be? How could she be called? Here, I suggest that she is a ‘gestational mother’ because she had that embryo in her body and provided it with oxygen and warmth, although they did not hit it in the usual way.^34^ It could therefore be assumed that ReIVF enables a double gestational motherhood by allowing two individuals to have/’carry’ the same entity in both their bodies. This is an extremely controversial assumption that needs to be thoroughly investigated from different perspectives, such as biology and philosophy. Although one may not agree with that, to consider such a possibility could be at least a useful exercise of imagination in view of the ‘proactive’ bioethics mentioned above. As Kendal has shown in her recent article, new technologies might lead to partial gestational motherhoods,^35^ and it cannot be excluded that ReIVF could be further developed in the future, so that an embryo or even a fetus could be first implanted in an individual’s body and then in another one’s.

## ‘Recombining’ biological motherhoods

Novel techniques like ReIVF and MST may divide biological motherhood as never before since they may not only create a split between a genetic motherhood and a gestational motherhood but also divide both genetic motherhood and gestational motherhood into smaller parts. While mitochondrial transfer techniques allow a double genetic motherhood since both two individuals can provide their eggs, with their related (mt)DNA, for the conception of an entity, techniques like ReIVF supposedly enable a double gestational motherhood, always following the assumptions given above.

If ReIVF allows a double gestational motherhood and MST allows a double genetic motherhood, these techniques may supposedly ‘recombine’ biological motherhood by joining its parts—that is genetic motherhood and gestational motherhood—together in two ‘complete’ biological mothers. For instance, based on a combination of ReIVF and MST, one may speculate that (1) both a first individual, ‘A’, and a second individual, ‘B’, provide eggs; (2) by using MST, the enucleated egg of ‘B’ has the nuclear material and, consequently, the nuclear DNA of ‘A’; (3) the egg of ‘B’ is combined with sperm in a device that is then inserted in the body of ‘A’; (4) once the fertilisation, incubation and the early embryo development have taken place within the body of ‘A’, the device is extracted; (5) after some time, the obtained embryo(s) is finally implanted in the body of ‘B’, who carries the embryo until childbirth. This potential scenario shows how a double genetic motherhood, enabled by MST, might be combined with a double gestational motherhood, supposedly enabled by ReIVF. In that way, the genetic and gestational dimensions of motherhood would no longer be separated, but they would be gathered in two distinct maternal figures that include both, that is in two ‘complete’ biological mothers.

## Ethical and social implications of combining ReIVF and MST

Although a combination of ReIVF and MST is unlikely to be applied because of its technical complexity, it is feasible and could be requested in the future. One reason why a couple should look for such a combination is that it can empower their reproductive autonomy by increasing their reproductive choices and, consequently, their reproductive freedom. Many types of couples such as queer couples may benefit from it since it provides them not only with another viable reproductive option but also with an opportunity that only couples in which both members can provide eggs and gestate could have: the opportunity to participate both genetically *and* gestationally to the creation of the child and, consequently, to have a unique connection with it.^28^

One of the benefits of combining ReIVF and MST is that it could help both the members of a couple smooth out the inequalities implied by ReIVF and MST. While ReIVF implies that an individual carries an entity for a greater amount of time than her partner, MST implies that who provides mtDNA gives a lower genetic contribution than who provides nuclear DNA since mtDNA represents only about 1% of the total genetic material.^36^ Thus, if techniques like ReIVF were combined with mitochondrial transfer techniques, the (perceived) inequalities between genetic mothers and gestational mothers could be reduced: on the one side, the mother who is less genetically connected with the offspring could carry it until childbirth, so that her weak genetic connection with the entity would be balanced by a stronger gestational connection; on the other, the mother who carries the entity for few hours or days could provide nuclear DNA so that her weak gestational connection with the entity would be balanced by a stronger genetic connection. This does not mean that both mothers are fully equal contributors to the creation of the child, but that they are certainly more equal in that creation since one’s weaker genetic relationship with the child is balanced by a stronger gestational relationship, and vice versa. This, coupled with the fact that no significative harm has been reported on the individuals involved in the use of ReIVF or MST, suggests that a combination of these techniques could be bioethically acceptable.

Although a combination of MST and ReIVF could be beneficial to many couples, there could emerge extremely challenging situations in which the biological motherhood of the same entity is disputed between many individuals. When ReIVF is combined with MST, it will no longer be a matter of only establishing which biological motherhood is more meaningful, whether genetic or gestational; rather, it will entail the need to deal with different genetic and gestational relationships. This could lead to a greater harmful competition for maternal status. Against ‘monomaternalism’, which establishes that there is always one and only one mother, it can be suggested that there are many ways to be(come) mothers, independently from biological or social relatedness (eg, foster mothers or walk-away mothers), and that every motherhood can be considered valuable.^37^ Thus, new techniques can help us redefine motherhood by widening the ways in which it can be perceived and even lived.

## Data Availability

No data are available.
